# A randomized, crossover pharmacodynamic study of immediate‐release omeprazole/sodium bicarbonate and delayed‐release lansoprazole in healthy adult volunteers

**DOI:** 10.1002/prp2.238

**Published:** 2016-05-19

**Authors:** Vijayalakshmi S. Pratha, Thomas McGraw, William Tobin

**Affiliations:** ^1^ Clinical Applications Lab., Inc.San DiegoCalifornia; ^2^ Merck & Co.KenilworthNew Jersey; ^3^ International HealthCare, LLCNorwalkConnecticut

**Keywords:** Crossover study, delayed‐release lansoprazole, efficacy, immediate‐release omeprazole/sodium bicarbonate, pharmacodynamics, safety

## Abstract

Proton pump inhibitors (PPIs) effectively block gastric acid secretion and are the treatment of choice for heartburn. PPIs differ, however, in onset of action and bioavailability. In this single‐center, open‐label, three‐way crossover study, onset of action of immediate‐release omeprazole 20 mg/sodium bicarbonate 1100 mg (IR‐OME) and delayed‐release (DR) lansoprazole 15 mg was evaluated in 63 healthy fasting adults. Subjects were randomized to once daily IR‐OME, or DR‐lansoprazole, or no treatment for 7 days. The primary efficacy endpoint was the earliest time where a statistically significant difference was observed between IR‐OME and DR‐lansoprazole in median intragastric pH scores for three consecutive 5‐min intervals on day 7. Secondary endpoints compared effects of active treatments on days 1 and 7 (e.g., time to sustained inhibition, percentage of time with pH >4). A significant difference in median intragastric pH favoring IR‐OME was observed on day 7 starting at the 10‐ to 15‐min interval postdosing (*P *=* *0.024) and sustaining through the 115‐ to 120‐min interval (*P *=* *0.017). On day 1, IR‐OME achieved sustained inhibition of intragastric acidity significantly faster than DR‐lansoprazole. IR‐OME maintained pH >4 significantly longer than DR‐lansoprazole over a 24‐h period (*P *=* *0.007) on day 7. Overall, results of this study demonstrate IR‐OME is safe and well tolerated and that treatment with IR‐OME results in significantly faster onset of action and better gastric acid suppression at steady state than DR‐lansoprazole.

**Abbreviations**
AE: adverse event
C_max_: maximum plasma concentrations
DR: delayed release
FDA: Food and Drug Administration
DR‐Lans: delayed‐release lansoprazole 15 mg
GERD: gastroesophageal reflux disease
H2RA: histamine‐2 receptor antagonists
IR: immediate release
IR‐OME: immediate‐release omeprazole 20 mg/sodium bicarbonate 1100 mg
LES: lower esophageal sphincter
OME: omeprazole
OTC: over‐the‐counter
PD: pharmacodynamic
PD‐E: pharmacodynamic evaluable
PK: pharmacokinetic
PPI: proton pump inhibitor
T_max_: time to achieve maximum plasma concentrations

## Introduction

Heartburn, the primary symptom of gastroesophageal reflux disease (GERD), is associated with gastric acid reflux into the esophagus, which can occur due to relaxation of the lower esophageal sphincter (LES) (Klauser et al. 1990). More than one third of adults in the United States experience heartburn at least once a month (Eisen 2001), and up to 11% of the general population experience it on a daily basis (Hunt 1999). Heartburn can be highly uncomfortable and can negatively impact quality of life; therefore, prompt relief is vital to patients’ well‐being (Lee et al. 2014). Acid suppression is the mainstay for the treatment of heartburn (Badillo and Francis 2014). Antisecretory agents that raise intragastric pH to >4.0 are effective in treating heartburn (Huang and Hunt 1999). A meta‐analysis of clinical trials involving patients with GERD and esophagitis showed a strong correlation (*P *<* *0.05; *r* = 0.87) between esophagitis healing rate at 8 weeks and duration of suppression of gastric acid secretion achieved over a 24‐h period (Bell et al. 1992).

Antacids, histamine‐2 receptor antagonists (H2RAs), and proton pump inhibitors (PPIs) increase intragastric pH to varying degrees through different mechanisms (Hunt 1999). PPIs offer the most effective and long‐lasting acid suppression compared with other medications (Badillo and Francis 2014; McRorie et al. 2014) and are well tolerated, making them the treatment of choice for heartburn (Attwood et al. 2015). PPIs are weak bases that accumulate selectively in the acidic space of the secretory canaliculus of stimulated parietal cells. Here, PPIs undergo acid‐dependent activation to form sulfenamide metabolites, which react with sulfhydryl groups of the gastric hydrogen potassium ATPase (H^+^/K^+^‐ATPase; i.e., proton pump). This interaction inactivates the proton pump and impedes the final step of gastric acid secretion by parietal cells independently of the nature of stimulation (Howden 1991). Because the sulfenamide metabolite forms an irreversible covalent bond with the proton pump, acid secretion is blocked until new enzyme molecules are synthesized, resulting in a prolonged duration of action (48–72 h) (Sachs et al. 1993; Shin and Sachs 2008). Optimal antisecretory effects of PPIs require the presence of acid, primarily through food‐induced stimulation of parietal cells and gastric acid secretion, and therefore, are dosed before meals to achieve maximal efficacy (Hatlebakk et al. 2000).

Patients with mild and uncomplicated symptoms of heartburn frequently self‐medicate with over‐the‐counter (OTC) medications (Haag et al. 2009), most frequently delayed‐release (DR) formulations of omeprazole (OME) and lansoprazole. DR‐PPI capsules are enteric coated because PPIs are acid labile and require protection from intragastric acid when taken orally (Howden 2005). The coating delays onset of action and may influence bioavailability, which varies across PPIs.

An immediate‐release (IR) formulation containing OME 20 mg and sodium bicarbonate 1100 mg without an enteric coating (IR‐OME, Zegerid^®^ capsules; Santarus Inc., San Diego, CA) was developed to mitigate coating‐related effects on absorption and improve onset of action. Sodium bicarbonate is a critical component of this formulation and plays a vital role in protecting OME from degradation, allowing rapid absorption of OME, leading to faster onset of antisecretory effect (Howden 2005). While the putative in vivo mechanism of sodium bicarbonate action remains theoretical, it is postulated that upon ingestion of IR‐OME, the sodium bicarbonate component causes a rapid rise in intragastric pH, providing a temporary stimulus for gastrin release. The rise in circulating gastrin levels stimulates parietal cells, allows for rapid uptake of circulating OME by the activated parietal cells, and leads to irreversible blocking of activated proton pumps (Howden 2005). Ingestion of uncoated OME without sodium bicarbonate results in a ~fivefold lower peak plasma concentration than OME with sodium bicarbonate (Howden 2005).

The US Food and Drug Administration (FDA) approved OME 20 mg/sodium bicarbonate 1100 mg for OTC use (Zegerid^**®**^ OTC; Bayer AG, Leverkusen, Germany) in December 2009 for treatment of frequent heartburn. Frequent heartburn is defined by the FDA as heartburn which occurs 2 or more days a week, and approved OTC products are indicated for 14‐day treatment of frequent heartburn, with patients permitted to repeat a 14‐day course every 4 months (Zegerid^®^ OTC [package insert] 2016).

This randomized, three‐way crossover pharmacodynamic (PD) study evaluated the earliest time to sustained difference in inhibition of intragastric acidity between the FDA‐approved OTC drugs – IR‐OME and DR‐lansoprazole (15 mg Prevacid^®^; Takeda Pharmaceuticals Inc., Deerfield, IL).

## Materials and Methods

### Study design

This single‐center, randomized, open‐label, no treatment‐controlled, three‐way crossover study (Protocol No. CL2008‐18, NCT01005719) was conducted in healthy adult volunteers in full accordance with the Declaration of Helsinki of 1975, as revised in 2008, concerning medical research in humans and complied with US 21 Code of Federal Regulations parts 50 and 56 concerning subject informed consent and institutional review board approval. The study was performed in accordance with standard operating procedures of Schering‐Plough HealthCare Products, Inc., and International Healthcare, LLC, to ensure adherence to Good Clinical Practices and protection of study subjects. Written informed consent was obtained from volunteers before the start of the study. The final protocol, amendments, and informed consent forms were reviewed and approved by the New England Institutional Review Board.

### Patients

Healthy non‐Asian adults aged 18–65 years who were free of any clinically significant disease that required a physician's care and/or would interfere with study evaluations, procedures, or participation were included in the study. Other key inclusion criteria were clinically acceptable physical examination, electrocardiogram, and laboratory tests (complete blood count with differential, serum chemistries, and urinalysis); women of child bearing potential had to agree to medically acceptable contraception throughout the study. Exclusion criteria included history of hypersensitivity, allergy, or intolerance to OME or other PPIs; history of peptic ulcer disease or other acid‐related gastrointestinal symptoms or heartburn with a frequency of >1 event per month; history (in the past year) suggestive of alcohol or drug abuse; gastrointestinal disorder or surgery that would lead to impaired drug absorption; participation in any study of an investigational treatment within 30 days of screening; participation in another study at any time during the study; positive *Helicobacter pylori* breath test at screening; and current/frequent user (or use within 14 days of first treatment administration) of antacids, OTC or prescription H2RAs, and PPIs.

### Randomization and treatment

**Figure 1:**
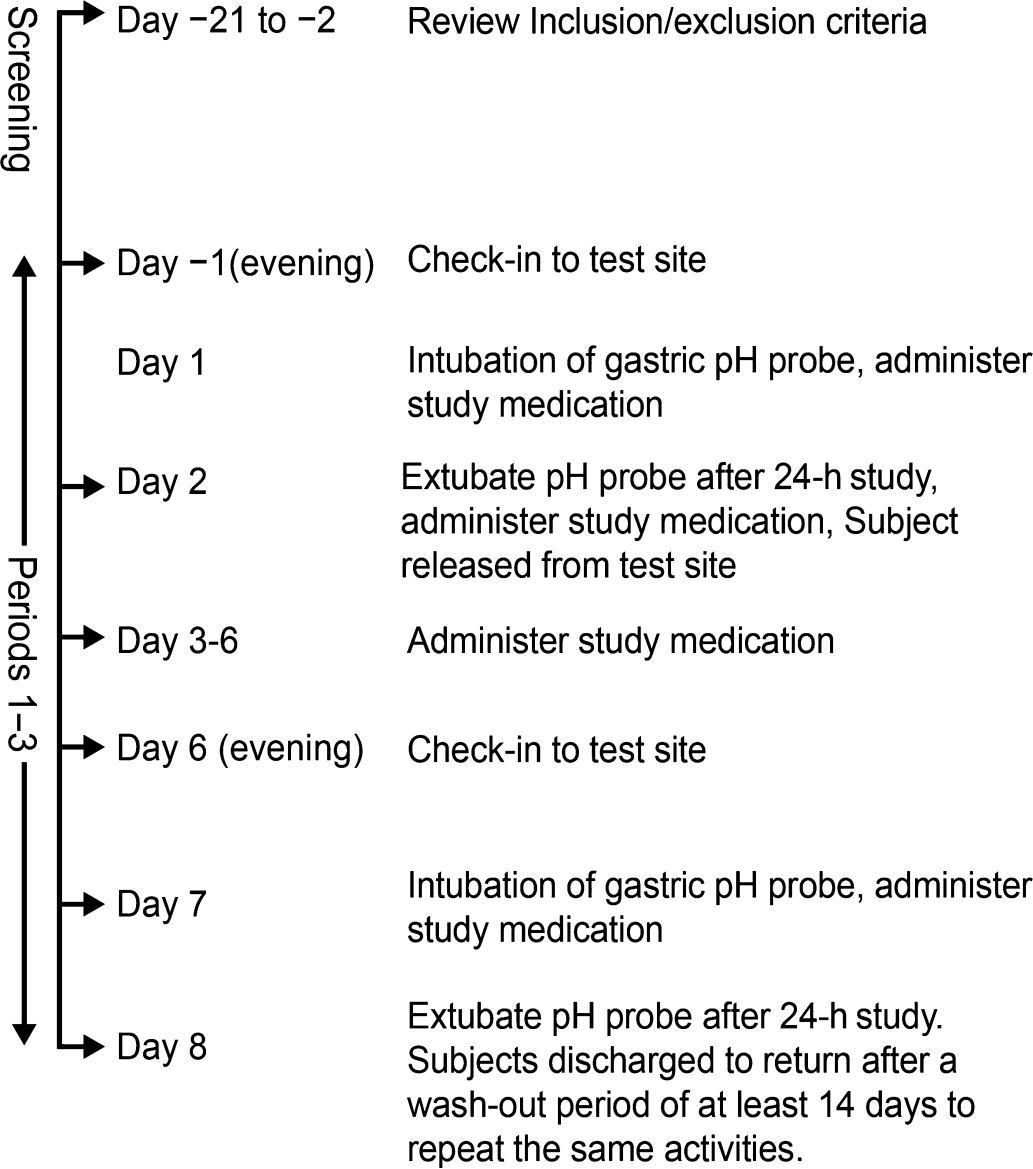
Study flowchart. Adverse events were assessed at screening and on all days of study period.

The study comprised a screening (day −21 to day −2) and three treatment (IR‐OME, DR‐lansoprazole, and no treatment) periods, each lasting 8 days (day −1 to day 8; Fig. 1). Subjects were randomized to receive each treatment based on subject number. A minimum 2‐week washout period occurred before crossover to another treatment. Administration of treatment occurred at the study site where, under supervision of site staff, intact capsules were taken orally in the morning on days 1–7 with 2 ounces of water approximately 1 h before a standardized breakfast.

Subjects randomized to no treatment received 2 ounces of water before breakfast. Subjects underwent a 24‐h intragastric pH study on the 1st and 7th days of each treatment period. This PD analysis was performed under fasting conditions. On the evening of day −1, subjects were provided a standardized evening meal and began a night‐time recumbent position at the study site. On the morning of day 1, after an overnight fast, subjects were intubated with the pH probe. The pH probe was inserted nasogastrically with the proximal probe 10 cm below the LES and held in place by taping it to the subject's face. The location of the LES was determined manometrically for each subject following a 5‐h fast at screening or any time before the first placement of the pH probe, if not located previously. Briefly, a pH probe with pressure sensors was placed at a depth of around 40 cm, and the distal location of the LES was determined on a graded withdrawal (by 1 cm) by the distance at which a rapid rise in pressure was noted. Within 2–3 cm, a change in the pressure was noted from a positive value in the stomach to a negative value as seen in the stomach. These findings were correlated to the pH changes noted at these locations from a gastric pH <2.0 to a pH 6.0 in the esophagus. Gastric pH was recorded every 4 sec. Approximately 1 h after intubation, the 24‐h pH recording began. Approximately 1 h after pH recording, subjects received the 1 dose of randomized treatments (IR‐OME, DR‐lansoprazole, or no treatment) with approximately 2 ounces of water. Ambulatory, continuous, 23‐h, postdose gastric pH monitoring was conducted during each period, with subjects mostly restricted to a recumbent position. On the morning of day 2 after bedtime fast, the pH probe was removed on completion of the 24‐h pH recording. Subjects received a single dose of assigned treatment approximately 1 h after extubation and were discharged from the study site. On the mornings of days 3 to 6, subjects returned for on‐site dosing and breakfast after an overnight fast. On day 6, subjects stayed overnight at the study site. On the morning of day 7, patients were intubated, had a pH recording, received a dose of assigned treatment, and were extubated 24 h later on day 8. Subjects were discharged from the study site after the 1st treatment period and returned to repeat the same activities after a washout period of at least 14 days. Subjects whose health or well‐being was adversely affected during the study and/or who failed to comply with protocol requirements were terminated from the study.

### Study endpoints

Primary endpoint was the earliest time at which a statistically significant difference was observed between IR‐OME and DR‐lansoprazole in median intragastric pH scores for three consecutive 5‐min intervals on day 7. Key secondary endpoints (between‐treatment comparisons) were time to sustained difference in inhibition of intragastric acidity between active treatments on day 1; comparison of effects between no treatment, IR‐OME, and DR‐lansoprazole at steady state (day 7 of treatment) for percentage of time with intragastric pH >4 over the 24‐h period.

Secondary endpoints (between‐treatment comparisons) also measured time to onset of inhibition of acid secretion on day 1 and day 7 of treatment, which was defined as first time to sustained median pH >3.5 for each of the 24 successive 5‐min periods (2 h) (the metric of pH 3.5 was based on the acid neutralization test specified in the FDA Antacid products for OTC human use monograph; Food & Drug Administration, 1974); early effectiveness of treatments at the beginning of steady state, which was defined as percentage of time with intragastric pH >4 during the first 4 h after treatment administration; comparison of effects of no treatment, IR‐OME, and DR‐lansoprazole at steady state (day 7 of treatment) for 24‐h median pH; and comparison of the two active treatments (subject‐wise time to sustained advantage over no treatment), which was defined as the earliest time during the first 4 h after dosing that median pH for the active treatment was >1 unit higher versus no treatment during the three subsequent 5‐min intervals.

Safety was assessed by evaluating the incidence, severity, and relationship of adverse events (AEs), laboratory test results, physical examination findings, and vital signs with study drug.

### Statistical analysis

All data summaries and analyses were performed using SAS release 9.1 or higher (SAS, Cary, NC). All statistical hypothesis tests were two sided and employed a level of significance of *α *= 0.05. For the primary analysis, comparisons between the two active treatments with respect to subject‐wise medians for each 5‐min interval were performed using a Wilcoxon signed rank test. Secondary endpoints relating to time to sustained difference in inhibition of intragastric acidity, and for each 5‐min period during the first 4 h posttreatment, were analyzed in the same manner. For additional secondary endpoints that were measured on a continuous scale, comparisons among study treatments employed a linear mixed model that included fixed factors, treatment, study period, and the random factor subject. Preliminary tests for the presence of carryover were performed employing a level of significance of *α *= 0.10. In cases of a statistically significant indication of possible carryover, the comparison of treatments was performed using the same linear model augmented with a factor adjusting for carryover. If an intragastric pH ≥3.5 was not met for any time point within the first 4 h after dosing, a duration of 240 min was imputed. All safety data were summarized using descriptive statistics and presented separately for each subject. AEs were coded using MedDRA version 10.1 (MedDRA MSSO, McLean, VA).

### Study populations

PD evaluable population comprised all subjects with valid data from all three treatment periods for at least one of the primary or secondary endpoints. PD population comprised all volunteers who received at least one dose of a study treatment and had at least one valid posttreatment evaluation of a primary or secondary endpoint. Safety population comprised all subjects who received at least one dose of a study treatment.

### Compliance with design and statistical analysis requirements

In this study, the inclusion and exclusion criteria were clearly defined, and all study populations (PD population, PD‐evaluable population, and safety population) had >5 patients. Subjects were randomized to receive each treatment with a minimum 2‐week washout period before crossover to another treatment. An open label design is commonly used for gastric acid suppression clinical trials (Rohss et al. 2002; Morelli et al. 2011; Ward et al. 2011; Shin et al. 2014) as the objective measurement of pH is not known to be prone to placebo effect or subject rating bias.

## Results

### Patient characteristics

**Figure 2:**
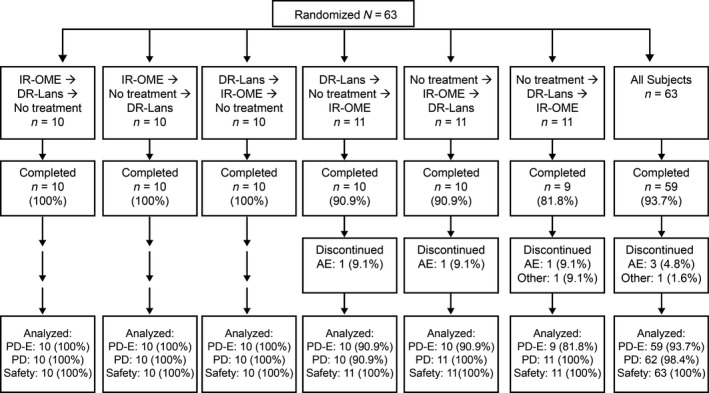
Subject disposition by treatment arm sequence. AE, Adverse event; DR‐Lans delayed‐release lansoprazole 15 mg; IR‐OME, immediate‐release omeprazole 20 mg/sodium bicarbonate 1100 mg; PD, pharmacodynamic; PD‐E, pharmacodynamic evaluable.

In total, 63 subjects were randomized to receive IR‐OME, DR‐lansoprazole, or no treatment per treatment‐arm sequence (Fig. 2); 59 (93.7%) subjects completed the study – three discontinued because of nontreatment‐related AEs, and the study sponsor discontinued one subject because of delayed completion of study procedures. PD population comprised 62 (98.4%) subjects, PD‐evaluable population comprised 59 (93.7%) subjects, and safety population comprised 63 (100%) subjects (Fig. 2). Efficacy results are shown for the PD‐evaluable population. The ages ranged from 18 to 48 years, and 55.6% of subjects were men (Table 1).

**Table 1:** Demographic and baseline characteristics, safety population (*N* = 63)

Characteristic	Subjects
Gender, *n* (%)	
Male	35 (55.6)
Female	28 (44.4)
Age (years), mean (SD)	27.6 (7.12)
Ethnicity, *n* (%)	
American Indian or Alaskan native	1 (1.6)
Black/African American	16 (25.4)
Native Hawaiian or Pacific Islander	9 (14.3)
White	37 (58.7)
Ethnic origin, *n* (%)	
Hispanic or Latino	27 (42.9)
Not Hispanic or Latino	36 (57.1)
BMI (kg/m^2^), mean (SD)	26.2 (4.74)
SBP (mmHg), mean (SD)	121.9 (7.10)
DBP (mmHg), mean (SD)	80.4 (6.28)

BMI, body mass index; DBP, diastolic blood pressure; SBP, systolic blood pressure; SD, standard deviation.

### Pharmacodynamic results

**Figure 3:**
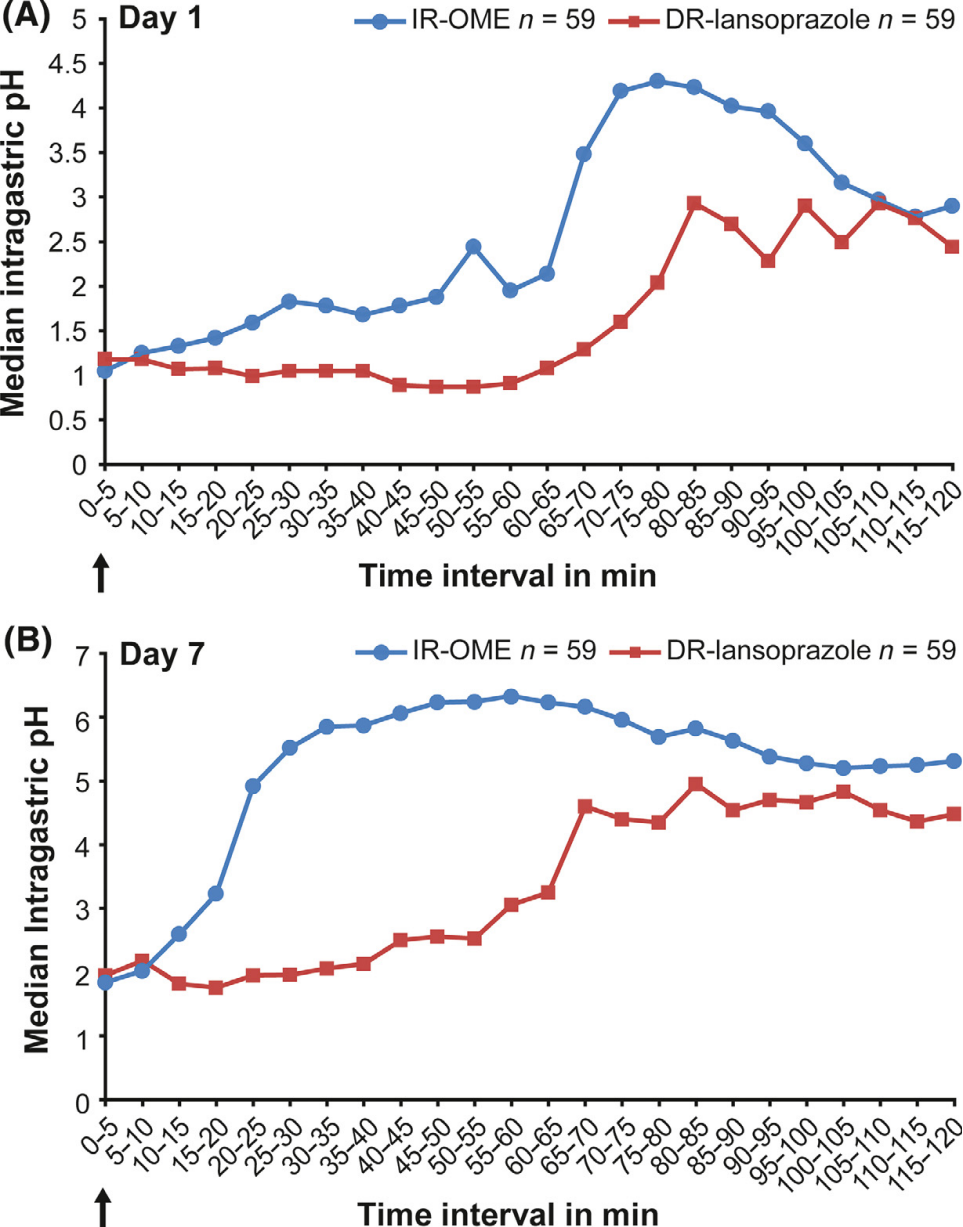
Median intragastric pH for the active study treatments over each 5‐min interval on day 1 (A) and on day 7 (B). The arrows indicate exact timing of administration of delayed‐release (DR) lansoprazole 15 mg or immediate‐release omeprazole 20 mg/sodium bicarbonate 1100 mg (IR‐OME).

The primary endpoint was met. Treatment with IR‐OME resulted in a significantly higher median intragastric pH than DR‐lansoprazole starting at the 10‐ to 15‐min interval (*P *=* *0.024) and continuing through the 115‐ to 120‐min interval (*P *=* *0.017) on day 7 (Fig. 3B).

**Figure 4:**
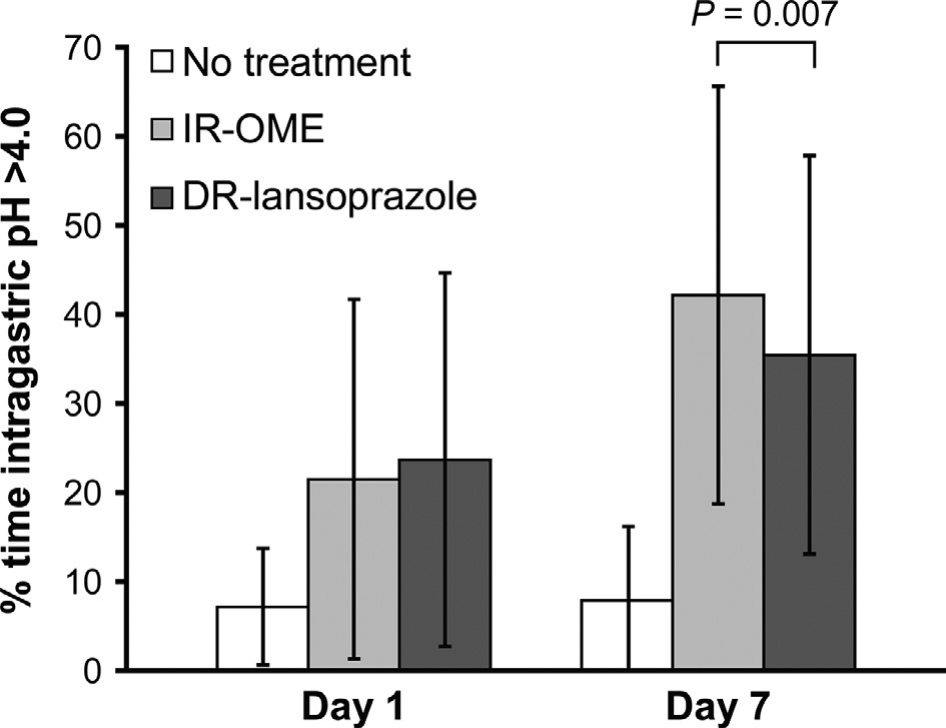
Mean percentage of time with intragastric pH >4 over 24‐h period following treatment with immediate‐release omeprazole 20 mg/sodium bicarbonate 1100 mg (IR‐OME) or delayed‐release (DR) lansoprazole or no treatment.

Results of secondary endpoints are shown in Figures 3A and 4, and Table 2. For median earliest time to sustained difference in median intragastric pH, treatment with IR‐OME resulted in statistically significant sustained inhibition of intragastric pH over each 5‐min interval from 10 to 95 min on day 1 (Fig. 3A). For mean percentage of time intragastric pH was >4 over a 24‐h period, treatment with IR‐OME resulted in a significantly better outcome than DR‐lansoprazole on day 7 but not on day 1 (Fig. 4). Treatment with IR‐OME also resulted in significantly better outcomes than DR‐lansoprazole on day 1 for mean time to achieve sustained advantage over no treatment, on day 7 for mean time to onset of inhibition of acid secretion, for median 24‐h intragastric pH, and on day 1 and day 7 for mean percentage of time intragastric pH was >4 during the first 4 h of dosing (Table 2).

**Table 2:** Summary of key secondary endpoints, PD‐evaluable population

	IR‐OME *n* = 59	DR‐lansoprazole *n* = 59	*P*‐value
Mean (SD) time in minutes to onset of inhibition of acid secretion			
Day 1	205.42 (71.66)	219.82 (48.21)	0.098
Day 7	111.86 (102.24)	152.71 (101.3)	0.0024
Mean (SD) percentage of time intragastric pH >4 during first 4 h of dosing			
Day 1	29.26 (25.57)	19.32 (21.32)	0.0021
Day 7	61.3 (31.32)	45.59 (32.79)	< 0.0001
Summary of median (min, max) 24‐h intragastric pH			
Day 1	1.31 (0.28, 5.80)	1.47 (0.35, 5.83)	0.31
Day 7	3.6 (0.69, 6.64)	3.10 (0.22, 6.58)	0.037
Number (%) of subjects with intragastric pH >4 more than 50% of the time			
Day 1	8 (13.6)	5 (8.8)	0.36
Day 7	26 (44.1)	18 (30.5)	0.021
Mean (SD) time in minutes to achieve sustained advantage over no treatment during the first 4 h after dosing			
Day 1	51.19 (60.95)	101.84 (77.58)	<0.0001
Day 7	23.98 (45.85)	35.17 (58.37)	0.20

DR‐lansoprazole, delayed‐release lansoprazole 15 mg; IR‐OME, immediate‐release omeprazole 20 mg/sodium bicarbonate 1100 mg; SD, standard deviation.

Post hoc analyses were performed to determine percentage of subjects with greater inhibition of intragastric acidity over the entire recording period. Results favored IR‐OME over DR‐lansoprazole at steady state on day 7 for the percentage of time pH was >4 or >3.5.

### Safety results

A total of 21 treatment‐emergent AEs were reported in 14 subjects (Table 3). One serious AE (acute appendicitis) was reported in a subject who received IR‐OME; the subject was discontinued from the study, and the serious AE was not considered related to study drug. No other medically significant AEs or deaths were reported during the course of the study.

**Table 3:** Treatment‐emergent adverse events (AEs), safety population (*N* = 63)

*n* (%)	IR‐OME *n* = 60	DR‐lansoprazole *n* = 61	No treatment *n* = 62	All subjects *N* = 63
Subjects with any treatment‐emergent AE	5 (8.3)	8 (13.1)	5 (8.1)	14 (22.2)
Nervous system disorders	2 (3.3)	5 (8.2)	4 (6.5)	8 (12.7)
Headache	2 (3.3)	4 (6.6)	4 (6.5)	7 (11.1)
Dizziness	0	1 (1.6)	0	1 (1.6)
Gastrointestinal disorders	2 (3.3)	3 (4.9)	0	5 (7.9)
Constipation	1 (1.7)	1 (1.6)	0	2 (3.2)
Flatulence	1 (1.7)	1 (1.6)	0	2 (3.2)
Vomiting	0	1 (1.6)	0	1 (1.6)
General disorders and administration site conditions	0	1 (1.6)	0	1 (1.6)
Influenza‐like illness	0	1 (1.6)	0	1 (1.6)
Infections and infestations	1 (1.7)	0	0	1 (1.6)
Appendicitis	1 (1.7)	0	0	1 (1.6)
Musculoskeletal and connective tissue disorders	0	1 (1.6)	0	1 (1.6)
Myalgia	0	1 (1.6)	0	1 (1.6)
Reproductive system and breast disorders	0	0	1 (1.6)	1 (1.6)
Dysmenorrhea	0	0	1 (1.6)	1 (1.6)
Respiratory, thoracic and mediastinal disorders	0	1 (1.6)	0	1 (1.6)
Pharyngolaryngeal pain	0	1 (1.6)	0	1 (1.6)

DR‐lansoprazole, delayed‐release lansoprazole; IR‐OME, immediate‐release omeprazole.

## Discussion

The IR‐OME formulation evaluated in the present study is known to heal severe reflux esophagitis, improve GERD symptoms such as heartburn (Orbelo et al. 2015), and has shown promise in mitigating a delayed onset of action (Howden 2005). The incorporation of sodium bicarbonate in the formulation of IR‐OME provides rapid buffering of gastric acidity thereby protecting the IR drug from inactivation. Pharmacokinetic (PK) analyses of 40 mg dose each of IR‐OME versus DR‐OME revealed higher maximum plasma concentrations (C_max_, 1019 vs. 544 ng/mL, respectively) and a significantly shorter time to achieve maximum plasma concentrations (T_max_, 25 vs. 127 min, respectively; *P *<* *0.01) (Howden 2005). Differences in the PK profile between IR‐OME and DR‐OME were confirmed in a comparative study, which showed that IR‐OME 20 mg was not bioequivalent to DR‐OME 20 mg because of higher maximal plasma levels and systemic exposure (Kearbey et al. 2013). The point estimate for the C_max_, AUC_t_, and AUC_inf_ ratios (IR‐OME 20 mg / DR‐OME 20 mg) were 220%, 117%, and 116%, respectively. The upper confidence limits for the C_max_ ratio exceeded the FDA threshold value of 125% for bioequivalence. In addition, a delayed absorption of DR‐OME (T_max_ >4 h) was also observed in a subset of subjects (Kearbey et al. 2013). The pH data presented in the current trial showing superior potency of IR‐OME over a DR‐lansoprazole formulation suggest that the superior C_max_ of IR‐OME might be an important parameter for potency of acid suppression.

The rapid onset of action was of particular relevance in the control of night‐time gastric pH in GERD patients with recurrent night‐time symptoms (Katz et al. 2007). After bedtime dosing with IR‐OME 40 mg for 7 days, median intragastric pH rapidly increased above 4 within 15 min of administration, and provided significantly faster and greater control of gastric acidity than DR‐lansoprazole 30 mg or DR‐esomeprazole 40 mg, during the first half of the night. Over the entire 8‐h night‐time period, gastric acid control was significantly better with IR‐OME than DR‐lansoprazole and was comparable to DR‐esomeprazole (Katz et al. 2007). Results of the present study reaffirm the more rapid antisecretory effect and sustained inhibition of gastric acid on day 1 and at steady state on day 7 with IR‐OME compared with DR‐lansoprazole.

PPIs have a short half‐life, and because not all gastric proton pumps are activated upon ingestion, achievement of steady‐state inhibition of acid secretion can take 2–3 days (Shin and Sachs 2008). A balance is achieved between inhibition of active proton pumps, subsequent stimulation of inactive gastric proton pumps after the drug has been eliminated from blood, and de novo synthesis of new gastric proton pumps. As expected, in the present study, IR‐OME demonstrated a significantly shorter onset of action than DR‐lansoprazole on day 1. The comparative advantage of IR‐OME on gastric acidity on day 1 may be attributed to sodium bicarbonate, which buffers gastric contents and turns on the proton pumps at a time when IR‐OME is available to bind proton pumps and inhibit acid production; this is evident because it reaches high plasma levels very quickly after oral administration, an effect not observed in the DR formulation without the bicarbonate.

This effect remains after steady state is reached on day 7, when a statistically significant difference in inhibition of gastric pH was observed with IR‐OME compared with DR‐lansoprazole. Likewise, a higher proportion of subjects receiving IR‐OME maintained intragastric pH >4 for a longer proportion of time over the 24‐h period compared to DR‐lansoprazole. These results augment those of a previous study comparing IR‐OME (40 mg), lansoprazole, and esomeprazole at steady state (Katz et al. 2007). Thus, rapid absorption of IR‐OME results in better 24‐h control of intragastric pH achieved even at the 20 mg dose. These factors – rapid onset of action coupled with extended acid suppression – are important differentiators of IR‐OME and DR‐lansoprazole and should be taken into consideration when choosing an OTC PPI for heartburn relief.

Further results of secondary endpoints in this study highlight the differences between IR‐OME and DR‐lansoprazole. The mean percentage of time intragastric pH was above 4 during the first 4 h of dosing was significantly higher on day 1, as well as on day 7. Although the time to achieve sustained advantage over no treatment during the first 4 h after dosing was significantly shorter for IR‐OME compared with DR‐lansoprazole on day 1, there was no significant difference in this parameter at day 7, indicating that both PPIs had reached steady state. However, at day 7, IR‐OME demonstrated significantly higher median 24‐h intragastric pH, and resulted in a significantly higher proportion of subjects with intragastric pH >4 more than 50% of the time compared with DR‐lansoprazole (both *P *<* *0.05). The percentage of time intragastric pH is over 4.0 in a surrogate of clinical efficacy since it directly correlates with clinically significant symptom relief and healing (Hunt 1995; Armstrong 2004). Therefore, while more extensive studies are required, our results point to the potential clinical superiority of IR‐OME. It should also be stated that as the intake of active drugs was followed by ingestion of food, the efficacy is independent of the food consumption and thus is probably independent of other factors known to affect reflux, such as gastric emptying.

The crossover design is the strength of this study because it aids in minimizing risk of bias and enables reliable comparisons between drugs. Despite the small sample size, results clearly indicate that IR‐OME provides significantly stronger acid suppression with a quicker onset and more sustained antisecretory effect. Results of the present study provide the framework for conduction of crossover studies enrolling adequate numbers of patients with heartburn to compare IR‐OME and DR‐lansoprazole using symptom relief as an efficacy endpoint.

The present study is not without limitations. Albeit typical, PD assessments were used for comparing potency between PPIs. PD studies involving continuous intragastric pH monitoring are widely used and have been the method of choice for comparing antisecretory potency among PPIs. PPIs inhibit acid secretion, limiting acid exposure to the esophagus, thereby providing symptomatic relief (van Pinxteren et al. 2010).

A study comparing DR‐OME with IR‐OME using heartburn relief as an efficacy endpoint showed IR‐OME was not more effective than DR‐OME despite the increased bioavailability and higher intragastric pH attained with the IR formulation (Walker et al. 2015). However, a few factors may have influenced this observation – substantial interindividual variability arising from inherent symptom severity of the individual and bias associated with symptom assessment (Walker et al. 2015).

Results of another study (Zheng 2009), which compared the efficacy of OME, pantoprazole, lansoprazole, and esomeprazole using symptom relief as an endpoint, were consistent with those of a study comparing the effects of the PPIs using intragastric pH as the endpoint (Rohss et al. 2004). Both studies concluded that DR‐esomeprazole 40 mg was more effective than DR‐omeprazole 20 mg, DR‐lansoprazole 30 mg, and DR‐pantoprazole 40 mg (Rohss et al. 2004; Zheng 2009). Therefore, whether a true relationship exists between relief of clinical symptoms and control of intragastric pH is unclear. Heartburn improvement positively correlates with quality of life; hence, symptom control is an important parameter to consider when evaluating efficacy. Both PD assessments, such as intragastric pH monitoring, and subjective assessments, such as symptom assessment, offer valuable information from a pharmacological and a clinical standpoint and should be weighted equally in a comparative evaluation of efficacy between PPIs.

In conclusion, results of this study demonstrate that compared to DR‐lansoprazole, IR‐OME had a significantly faster onset and duration of gastric acid suppression. Furthermore, results are consistent with previous studies showing IR‐OME and DR‐lansoprazole are safe and well tolerated.

## Author Contributions

Pratha, McGraw, and Tobin participated in research design, conducted experiments, performed data analysis, and wrote or contributed to the writing of the manuscript. All authors have read and approved the final manuscript to be submitted.

## Disclosures

V. P. served as a principal investigator and received stipend for the conduct of the study. T. M. was employed by Merck & Co., Inc., Kenilworth, NJ, USA at the time of the study. W. T. has no conflicts to declare.
